# New PAM Improves the Single-Base Specificity of crRNA-Guided LbCas12a Nuclease

**DOI:** 10.3390/life12111927

**Published:** 2022-11-18

**Authors:** Mariia A. Misiurina, Angelina V. Chirinskaite, Aleksandra S. Fotina, Andrey A. Zelinsky, Julia V. Sopova, Elena I. Leonova

**Affiliations:** 1 Center of Transgenesis and Genome Editing, St. Petersburg State University, Universitetskaja Emb., 7/9, 199034 St. Petersburg, Russia; 2 Laboratory of Amyloid Biology, St. Petersburg State University, Universitetskaja Emb., 7/9, 199034 St. Petersburg, Russia

**Keywords:** LbCas12a, protospacer adjacent motif, collateral activity, genotyping

## Abstract

The RNA-guided Cas12a nuclease forms a complex with a CRISPR RNA (crRNA) to cleave the double-stranded DNA target. Among others, Cas12a protein from *Lachnospiraceae bacterium* (LbCas12a) is widely used for biomedical research. For target recognition, LbCas12a requires a specific nucleotide sequence, named a protospacer adjacent motif (PAM). Besides the canonical TTTV PAM, LbCas12a can recognize other suboptimal PAMs. We examined a novel TTAA PAM for the LbCas12a nuclease and found that the specificity of cleavage was increased. We found that single nucleotide substitutions at all positions of the guide RNA except the 20th position blocked the cleavage of the target DNA. The type of nucleotide substitutions (U-A, U-C or U-G) did not affect the efficiency of cleavage in the 20th position. When we used the canonical PAM under the same conditions, we observed the cleavage of target DNA by LbCas12a in many positions, showing less specificity in given conditions. The efficiency and specificity of the LbCas12a nuclease were evaluated both by gel-electrophoresis and using FAM-labeled single-stranded probes. We were able to assess the change in fluorescence intensity only for several variants of guide RNAs. High specificity allows us to type single nucleotide substitutions and small deletions/insertions (1–2 nucleotides) and look for target mutations when knocking out.

## 1. Introduction

CRISPR (Clustered Regularly Interspaced Short Palindromic Repeats) are components of the adaptive immune system in bacteria and archaea. The CRISPR–Cas genomic loci consist of short palindromic repeats interspaced by unique sequences originating from foreign genetic elements. These sequences named “spacers” get transcribed into small CRISPR RNA (crRNA) and form a complex with Cas proteins to guide the nuclease to complementary foreign nucleic acids (protospacers) for cleavage [1]. Cas12a ribonucleoprotein complex comprises crRNA and Cas12a protein (also known as Cpf1) and, unlike Cas9, CRISPR systems associated with Cas12a process crRNAs without the requirement for additional trans-activating crRNA (tracrRNA) [2]. The mature crRNA consists of 42–44 nucleotides including 19 nucleotides direct repeat which forms a short hairpin followed by 23–25 nucleotides of a spacer sequence (or guide sequence). The previous findings also demonstrated that the *Francisella novicida* Cas12a (FnCas12a) cleaved the target DNA using only the 20-nt guide-containing crRNA [3,4]. Cas12a nuclease has bilobed molecular architecture and contains two major parts: a nuclease lobe (NUC) and an alpha-helical recognition lobe (REC). The REC lobe consists of two domains: REC1 and REC2, which have been shown to coordinate the crRNA-target DNA heteroduplex. The NUC lobe is composed of the RuvC domain and additional domains: PI, WED, Nuc and bridge helix (BH) [5]. The RuvC-like endonuclease domain of Cas12a is subdivided into three motives (RuvC-I–III) that form the endonuclease active center, but it lacks the second HNH endonuclease domain unlike Cas9 protein [3]. 

The mechanisms of cleavage activity for Cas9 and Cas12 are significantly different: Cas9 cleaves the target strand (TS) and non-target strand (NTS) with the HNH and the RuvC domain, respectively, while Cas12 employs only the RuvC domain that performs the cleavage of the target DNA strands [6]. Some previous biochemical studies suggested that Cas12a contained two DNA nuclease active sites and cleavage of the non-target DNA strand by the RuvC domain was a prerequisite for target strand cleavage by the Nuc domain [5]. However, extensive mutational analysis revealed that both the target and non-target DNA strands were cleaved by the same catalytic mechanism in a single active site RuvC in Cas12a enzymes [7]. Cas12a has demonstrated nickase activities on mismatched double-stranded DNA (dsDNA) targets in positions 12–13 and 14–15 in gRNA [8].

In contrast to Cas9 that recognizes G-rich PAM (Protospacer Adjacent Motif), the catalytic activity of Cas12a requires recognition of the T-rich PAM sequence located at the 5’-end of the protospacer. Cas9 nuclease activity leads to a double-stranded break and a formation of blunt ends or ends with a 1 nucleotide overhang. Cas12a generates a PAM-distal double-stranded DNA break with a 5’-overhang of 4 or 5 nucleotides that leads to a formation of the products with sticky ends. 

A fundamental property of Cas12 nucleases is their ability to cleave the non-specific single-strand DNA (ssDNA) molecules (also known as trans-cleavage or collateral activity). Previous studies showed that the binding of crRNA to target DNA activated Cas12a for both cleavages of site-specific double-stranded DNA molecule (dsDNA) and non-specific ssDNA [6]. In addition, Cas12a has both crRNA-directed sequence-specific nicking activity and nonspecific nicking activity [9]. The binding of the target dsDNA is initiated by the recognition of the PAM sequence. After cleavage of the target dsDNA, the PAM-distal cleavage product is released, and the PAM-proximal dsDNA remains bound to the Cas12a-crRNA complex. This maintains Cas12a in a catalytically activated conformation, allowing for nonspecific cleavage of ssDNA (single-stranded DNA). This attribute is known as “collateral effect” or trans-cleavage activity [8].

Mutation K538R in LbCas12a from *Lachnospiraceae bacterium* leads to significantly lower cutting efficiency at CTTV, TTCV, and TCTV PAMs. K538 was found to be heavily involved in the interaction with the PAM complementary strand [10].

Many platforms for the detection of nucleic acids have been developed based on the trans-cleavage property of Cas12a nuclease. In 2019, Yifan Dai et al. reported an electrochemical biosensor to detect viral nucleic acids, including human papillomavirus 16 (HPV16) and parvovirus B19 (PB-19) [11]. Recently the system CANTRIP which combines two detection platforms consisting of CRISPR-Cas12a and fluorescent copper nanoparticles into a single reaction has also been demonstrated as a promising diagnostic tool [12]. Similar to Cas9, Cas12a can be reprogrammed to genome editing. To apply this system as a universal biotechnological tool, the binding of crRNA to the target DNA sequence should be specific while the nucleotide mismatches between the crRNA and the target sequence could lead to the lack of catalytic activity of the nuclease [13]. Another limitation in applying Cas12a is its relatively low efficiency for human genome editing [14].

In addition, broad application of CRISPR-Cas12a can be limited by the relatively rare occurrence of their T-rich PAM sequence in mammalian genomes [15]. The LbCas12a PAM consensus sequence is a TNTN motif, but nuclease can also recognize TACV, TTCV, CTCV and CCCV motifs [15]. Insofar as the searching for a new and more frequent PAM sequence is still ongoing, we demonstrated a PAM sequence for LbCas12a that has not been described previously. To expand the possibilities of using Cas12a for genomic editing and diagnostic testing we focused on the study of a previously undescribed PAM for Cas12a-TTAA.

In the presented study, we used fluorescence analysis and gel-based experiments to determine functional activities and single-base specificity of LbCas12a by introducing single nucleotide substitutions into the guide RNA sequence, using new TTAA PAM. Further, we studied the efficiency of the new PAM on LbCas12a nuclease activity to design a test system for genotyping. As a target DNA, we have selected a region of the pde6b (Phosphodiesterase 6B) gene of the *Mus musculus* carrying a double-nucleotide deletion.

## 2. Materials and Methods

### 2.1. Expression and Purification of LbCas12a

LbCas12a protein was expressed and purified according to [6] with modifications. pMBP-LbCas12a was a gift from Jennifer Doudna (Addgene plasmid #113,431; http://n2t.net/addgene:113431 accessed on 13 November 2022; RRID: Addgene_113431). *E. coli* strain BL21(DE3) containing pMBP-LbCas12a expression plasmid was grown in Terrific Broth at 16 °C for 14 h. Cells were harvested and resuspended in Lysis Buffer (50 mM Tris-HCl, pH 7.5, 500 mM NaCl, 5% (*v*/*v*) glycerol, 1 mM DTT, 0.5 mM PMSF and 0.25 mg/mL lysozyme), disrupted by sonication, and purified using Bio-Rad NGC (Bio-Rad, Hercules, CA, USA) on Ni-NTA column (GE, Marlborough, MA, USA). After overnight TEV cleavage at 4 °C, protein solution was again loaded on the Ni-NTA resin, and unbound LbCas12a protein was collected and transferred to storage buffer containing 20 mM Tris-HCl, pH 7.5, 200 mM NaCl, 1 mM DTT and 5% (*v*/*v*) glycerol). 

### 2.2. DNA Target and crRNA Preparation

A fragment of the *Mus musculus* Exonuclease 1 gene (*Exo1*) was used as a substrate for studying the activity and specificity of the LbCas12a nuclease. Genomic DNA was extracted using GeneJET Genomic DNA Purification Kit (Thermo Fisher Scientific, Waltham, MA, USA). DNA target (395 bp) was amplified with Phusion High-Fidelity DNA Polymerase (Thermo Fisher Scientific, Waltham, MA, USA). Gel extraction and purification of DNA target was performed using GeneJET gel extraction kit (Thermo Fisher Scientific, Waltham, MA, USA).

For all variants of crRNAs, DNA template fragments were amplified using Tag DNA polymerase (Evrogen, Moscow, Russia). The forward primer contained the promoter sequence for bacteriophage T7 RNA polymerase and the conserved crRNA sequence, and the reverse primers contained the conserved crRNA sequence and a guide sequence to the selected region of the target DNA with or without mutations. PCR products were incubated with T7 RNA polymerase (Thermo Fisher Scientific, Waltham, MA, USA) for 1.5 h at 37 °C. To remove DNA template 2 µL (2 U) of DNase I (Thermo Fisher Scientific, Waltham, MA, USA), was added and incubated at 37 °C for 15 min. DNase I was inactivated by heating at 65 °C for 10 min.

All oligonucleotide sequences used in this study are available in Table S1.

### 2.3. In Vitro dsDNA Cleavage Assay

CrRNA variants (880 ng/μL) was assembled with LbCas12a (96.7 ng/μL) in the cleavage buffer contained 100 mM Tris-HCL (pH 8.8), 500 mM KCL, 15 mM MgCl_2_, 0.8% Nonidet P40. After incubation for 10 min at 37 °C dsDNA substrate (6.4 ng/μL) was added and kept for 20 min at the same temperature. After incubation 0.5 μL of Proteinase K enzyme (Thermo Fisher Scientific, Waltham, MA, USA), was added and mixture was incubated for 10 min at 37 °C. The efficiency of dsDNA cleavage was assessed in 1.7% agarose gel pre-stained with Ethidium bromide (Panreac Applichem, Darmstadt, Germany). The presence of cleavage products of 226 and 168 bp in size was evaluated in 1.7% agarose gel. The rate of DNA cleavage was calculated as ((A − Ao) + (B − Bo)/((A − Ao) + (B − Bo) + (C − Co) × 100%, where A and B is the intensity of the band corresponding to two DNA cleavage product; C is the intensity of the RNA band, Ao, Bo, Co are the background values for the corresponding quantities. The intensity of bands was evaluated using Image Lab 6.0.1.

### 2.4. Fluorescence Analysis

CrRNAs were assembled with LbCas12a as described above. The reaction was initiated by adding labeled ssDNA reporters in concentration 5 or 0.5 pM/μL. The relative fluorescence was measured on Bio-Rad CFX96 Touch System (Bio-Rad, Hercules, CA, USA), Point 0 was taken, incubated for 30 min at 37 °C, and then the end point was taken. The difference between the fluorescent signal value of the end point and point 0 was plotted. The cut-off line (2000 RFU) was calculated as Negative Control Average + Tolerance. Tolerance was set at 20% of the total RFU range, and values below this line are considered as negative.

### 2.5. Genotyping

A fragment of the *Mus musculus* Phosphodiesterase 6B (*Pde6b*) gene was chosen as a target. For this, genomic DNA from 3 wild-type (wt) mice and 3 mice with a two-nucleotide deletion in the selected fragment (KO) was isolated. A 468 bp fragment was generated from the outer primers. Next, using internal primers, the sequence of the new PAM was introduced and the target fragment of 124 bp was obtained.

The purified fragment was used to assess double-stranded target DNA cleavage and LbCas12a collateral activity. Guide RNA was obtained using in vitro transcription. The spacer region of this RNA was fully complementary to the wild-type target DNA fragment.

## 3. Results

### 3.1. Newly Described PAM for LbCas12a Influences the Substrate DNA Recognition

It was previously shown that LbCas12a exhibits different cleavage activity on various PAM sequences [15]. Canonical PAM for LbCas12a considered to be TTTV, but non-canonical PAMs with decreased efficiency, such as CTTV, TCTV, and TTCV, were reported [16]. We have identified the new PAM sequence (TTAA) and compared the activity of the LbCas12a nuclease in the presence of our new PAM1 (TTAA) with canonical PAM2 (TTTA). We choose the canonical TTTA PAM sequence, which conforms to the consensus 5′ TTTV-3′ PAM [17]. Moreover, the interaction of LbCas12a and TTTA PAM has been well characterized in crystal structures [16,18].

**Figure 1:**
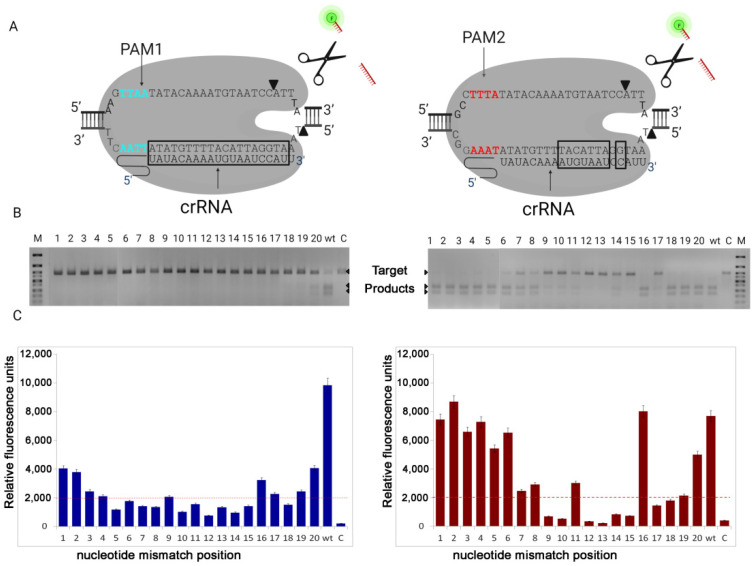
(**A**) Nuclease LbCas12a in complex with crRNA and DNA target (*Exo1*), containing two variants of PAM sequences: PAM1(TTAA) and PAM2(TTTA). Nucleotides intolerant to mismatches in crRNA are in black frames. (**B**) Electrophoretic diagram of the dsDNA cleavage shows that cleavage efficiency depends on PAM sequence and mismatched nucleotide position. (**C**) Non-specific ssDNA degradation (collateral activity), measured by reporter fluorescence intensity, correlates with dsDNA cleavage efficiency. Red dotted line designates the cut-off line, values below this line are considered as negative.

We have obtained a series of crRNAs consisting of a 20-nt spacer sequence complementary to a DNA fragment of the *Mus musculus* gene Exonuclease 1 (*Exo 1*). We assembled LbCas12a with 20 variants of crRNA containing single nucleotide substitutions in positions 1–20 of spacer region (Figure 1A, Table S1) and used this complex in reaction with two target sequences differing only in PAM sequence: TTAA (PAM1) and TTTA (PAM2). To measure the intensity of LbCas12a cleavage, amplified fragments of the double-stranded DNA target (395 bp of the *Exo1* gene) containing the PAM1 and PAM2 sequences, respectively, were used. As a positive control, the samples contained crRNA without substitutions (wt), as a negative control, the reactions did not contain crRNA (C). For each DNA target, reactions were performed in three replicates. The results were evaluated as efficiency of dsDNA target cleavage and non-specific ssDNA degradation (collateral activity), measured by reporter fluorescence intensity. Fluorescence intensity after incubation with LbCas12a, guide RNAs with base-pair mismatches at positions 1–20, and *Exo1* target DNA containing PAM1 or PAM2 was measured by end point. To separate samples with collateral activity from the rest the cut-off value was calculated according to the instruction manual for Bio-Rad CFX Maestro Software 2.0 (Bio-Rad, Hercules, CA, USA). 

Each reaction was performed by using different guide RNA variants carrying one base-pair mismatch at positions 1–20, respectively. As a negative control (C), sample without crRNA was incubated at the same conditions (Figure 1). The (wt) probe contains guide RNA without any mismatches. To analyze the intensity of cleavage of DNA target, we used the electrophoresis in agarose gel.

After incubation of LbCas12a with an amplified *Exo1* DNA fragment containing the new PAM TTAA (PAM1), we observed 100% cleavage activity only for the samples with crRNA without any mismatches (wt). Additionally, we found the presence of a small amount of cleavage products in the samples with base-pair mismatches at position 20. In other cases, in contrast, no DNA cleavage was observed. The collateral activity of LbCas12a in samples containing PAM1 was observed only for guide RNA without base-pair mismatches (wt) that corresponds to the results of electrophoresis. This indicates a high specificity of double-stranded DNA cleavage for LbCas12a, which recognizes the new PAM1, although with reduced efficiency. 

After LbCas12a was incubated with an amplified *Exo1* DNA fragment, that contains a fragment of the canonical PAM TTTA (PAM2), we observed that 100% cleavage occurred in wild-type samples and in samples with base-pair mismatches at positions 1–5 and 18–20. In contrast, in samples with mismatches at positions 12, 13, and 15, DNA cleavage was not observed at all. In the remaining samples with base-pair mismatches partial cleavage was observed with varying intensity (Figure 1). After incubation with a DNA target containing PAM2, the intensity of the fluorescent signal corresponded to the electrophoresis data, except for samples with base-pair mismatches at positions 17–19. This may be due to the fact that the lack of complementarity between sgRNA and target DNA at these positions leads to inhibition of LbCas12a catalytic activity or its significant decrease. However, the mutation located on the last PAM distal position 20 slightly maintained a catalytically active state of nuclease. The obtained data indicate the lower specificity of LbCas12a, which recognizes PAM2, although high efficiency. 

**Figure 2:**
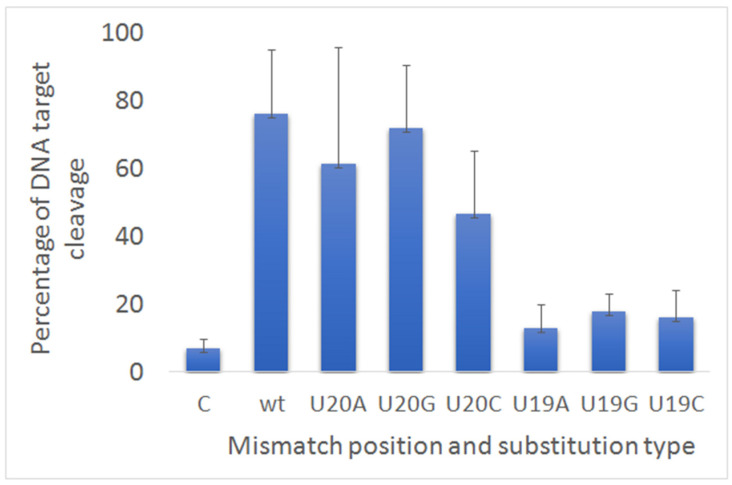
The cleavage activity LbCas12a after incubation with dsDNA target and guide RNAs carrying substitutions with different nucleotides.

We explored if substitution in guide RNA with different types of bases could influence the LbCas12a cleavage of DNA target containing the new PAM1. In previously described experiments, as a result of a mutation in positions 20 and 19 of crRNAs, uracil was replaced by adenine (U20A and U19A). We additionally obtained variants of crRNAs with substitutions of uracil to cytosine (U20C) and guanine (U20G) at position 20 and at position 19 (U19C, U19G) (Figure 2). We chose these two positions, which had different substrate digestion efficiency. In position 20, splitting was effective, and in position 19 it was weak. As a result, we could not reveal any statistically significant differences of DNA cleavage between samples with substitutions on bases with adenine, cytosine or guanine for both 19 and 20 positions of crRNA. Therefore, different types of substitutions in 3′ terminal end of crRNA did not influence the LbCas12a activity.

### 3.2. Newly Described PAM for LbCas12a Can Be Used in Genotyping Experiments

**Figure 3:**
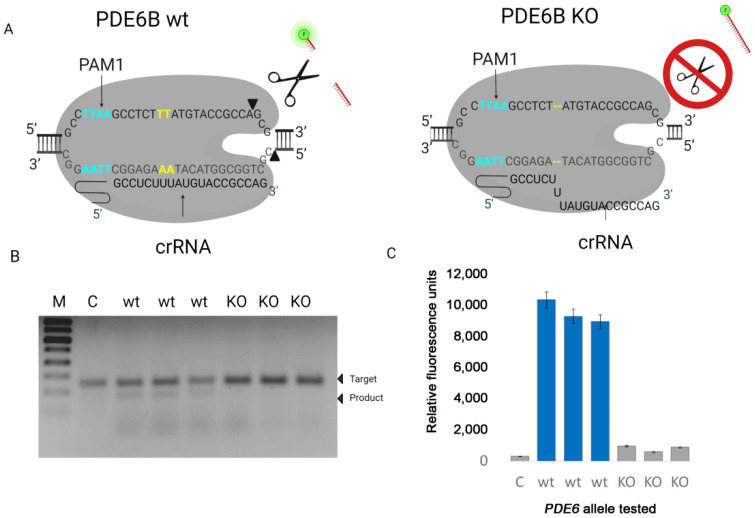
(**A**) Complex of target DNA PDE6B (wt) with LbCas12a and guide RNA (crRNA). (**B**) The cleavage efficiency of nuclease LbCas12a on dsDNA target WT and carrying a deletion (KO). The control sample (C) did not contain guide RNA. (**C**) The collateral activity of nuclease LbCas12a on dsDNA wt target and target, carrying a deletion (KO). The control sample (C) did not contain guide RNA.

Next, we tested the activity of LbCas12a on DNA target containing 2-nucleotide deletion and PAM1. We obtained the DNA target with the deletion of 2 nucleotides from the tissue of Pde6b-KO mice, which we obtained in our laboratory using the CRISPR/Cas9 system (data not published). A spacer region of crRNA was designed to recognize dsDNA target Pde6b from wt mice. The ability of LbCas12a to detect deletion was evaluated of dsDNA target cleavage by gel electrophoresis, single-molecule fluorescence analysis, and gel-based experiments (Figure 3). 

Based on our data, the presence of a two-nucleotide deletion in the studied target DNA leads to inhibition of LbCas12a activity, which can be detected using gel electrophoresis and fluorescent analysis. As can be seen from the presented results, we observed the presence of cleavage products only in samples containing wild-type target DNA.

**Figure 4:**
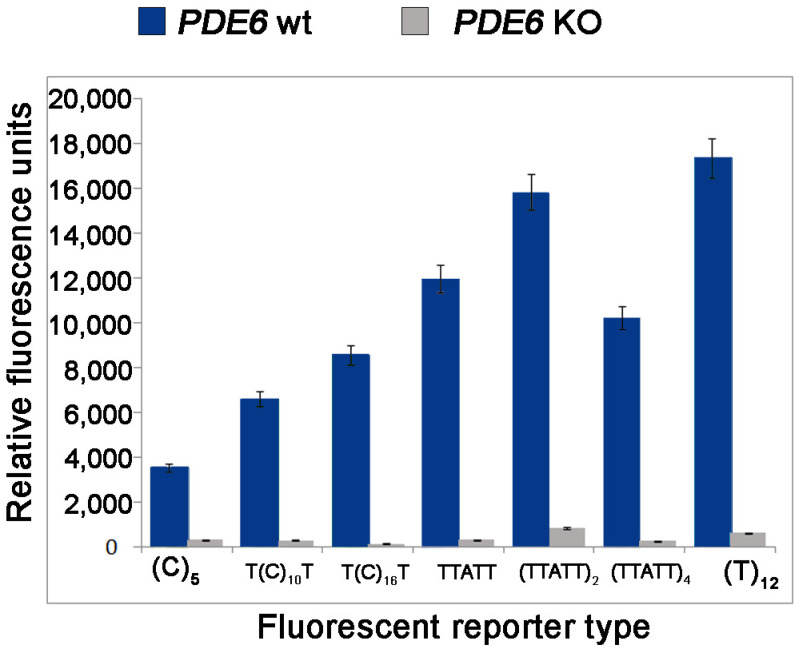
The presence of a fluorescent signal with the use of different types of reporters depending on the presence of the deletion in the *Pde6b* gene. The fluorescence intensity value is estimated from the end point.

Next, we studied the specificity of collateral activity for LbCas12a guided to Pde6b DNA target containing the PAM1 with a fluorophore quencher (FQ)-labeled reporter assay using FAM-labeled ssDNA reporter as a substrate (Figure 4). It was revealed that LbCas12a guided to recognize PAM1 sequences demonstrated different levels of activity depending on the length of ssDNA reporter. A significant increase in fluorescence was observed for LbCas12a aimed at recognizing PAM1 using 12nt reporter. We made our choice of a reporter carrying a fluorescent label FAM (designation on the graph (TTATT)_4_ based on the optimal combination of fluorescent signal/noise. This reporter can be used in subsequent tests, including for assessing the minimum concentration of target DNA that can be detected by the platform we developed.

## 4. Discussion

Taken together, our results demonstrate the ability of the LbCas12a nuclease to recognize the new PAM TTAA sequence, although its DNA target cleavage activity is reduced. Our experiments showed that even with the use of sgRNA without any substitutions (wt) the LbCas12a was unable to completely cleave the DNA with the new PAM1, while under the same conditions, the nuclease completely cleaved DNA with canonical PAM2. When we used sgRNA containing base-pair mismatches to cleave DNA with PAM1, only in a single sample (mismatch at 20th position) we found a barely noticeable cleavage. It should be noted that, in this case, the results of electrophoresis fully correspond to the data of fluorescent analysis, what cannot be said about reactions using DNA target with canonical PAM2. We also were surprised to observe that with base-pair mismatches at positions 18 and 19, we observed complete cleavage of the DNA target on electrophoresis, but there was no collateral activity on fluorescent analysis. More research is needed to understand this phenomenon. We assume that this is due to the fact that complementarity at these positions is important for the activation of the nuclease collateral activity center.

Next, we decided to test whether the nucleotide substitution affects the cleavage of DNA target with PAM1. For this, two samples were taken: one with a base-pair mismatch in position 20, where we observed weak cleaved, and the second with a base-pair mismatch in position 19, where there was no cleavage. As we described above substitutions of nucleotides at position 20 (U20A, U20C, U20G) and at position 19 (U19A, U19C, U19G) in the guide RNA did not change the results of the cleavage. From what follows, that the composition of the nucleotides does not affect the activity of the nuclease.

Based on the obtained data, we concluded that the use of the new PAM1 significantly increased the specificity of the nuclease, despite the decrease in its activity and a narrowing of the target selection window. This, in turn, opens up broad prospects for the development of various LbCas12a-diagnostic systems, particularly, the development of a test system for fast genotyping of mice carrying point mutations and microdeletions. The detection of such mutations using PCR is difficult; therefore, it is necessary to carry out numerous sequencings of the obtained DNA samples and analyze complex chromatograms using special programs. Because LbCas12a directed on PAM1 is sensitive to single mismatch in guide RNA, it can be used not only for genotyping, but also for detecting viral infections in clinical practice.

The collateral activity of the nuclease depends on the length of the quencher (FQ)-labeled reporter, with it being more active on FAM-labeled AT-rich substrates. This is partially consistent with previously reported data by [19]. Surprisingly, 20-nt reporter was less effective than 12-nt reporter, and ROX-labeled C-rich reporters were less efficient than FAM-labeled T-rich reporters of similar length. Overall, our results establish that high specificity of the LbCas12a directed to the new PAM (TTAA) can be useful for the broadening of genome engineering applications. Our findings have led to a better understanding of Cas12a nuclease activity, which could potentially help in the development of improved diagnostic platforms for both rapid genotyping of genetically modified animal lines and the diagnosis of various diseases.

## Data Availability

Not applicable.

## Supplementary Materials

The following supporting information can be downloaded at: https://www.mdpi.com/article/10.3390/life12111927/s1; Table S1: Oligonucleotides used in this work.
